# Bioproduction of testosterone from phytosterol by *Mycolicibacterium neoaurum* strains: “one-pot”, two modes

**DOI:** 10.1186/s40643-022-00602-7

**Published:** 2022-11-04

**Authors:** Daria N. Tekucheva, Vera M. Nikolayeva, Mikhail V. Karpov, Tatiana A. Timakova, Andrey V. Shutov, Marina V. Donova

**Affiliations:** grid.470117.4 G.K. Skryabin Institute of Biochemistry and Physiology of Microorganisms, Federal Research Center “Pushchino Center for Biological Research of the Russian Academy of Sciences”, Prospect Nauki 5, Pushchino, Moscow Region 142290 Russia

**Keywords:** Testosterone, Phytosterol, *Mycolicibacterium neoaurum*, “One-pot” bioproduction, 17β-hydroxysteroid dehydrogenase

## Abstract

The main male hormone, testosterone is obtained from cheap and readily available phytosterol using the strains of *Mycolicibacterium neoaurum* VKM Ac-1815D, or Ac-1816D. During the first “oxidative” stage, phytosterol (5–10 g/L) was aerobically converted by Ac-1815D, or Ac-1816D to form 17-ketoandrostanes: androstenedione, or androstadienedione, respectively. At the same bioreactor, the 17-ketoandrostanes were further transformed to testosterone due to the presence of 17β-hydroxysteroid dehydrogenase activity in the strains (“reductive” mode). The conditions favorable for “oxidative” and “reductive” stages have been revealed to increase the final testosterone yield. Glucose supplement and microaerophilic conditions during the “reductive” mode ensured increased testosterone production by mycolicibacteria cells. Both strains effectively produced testosterone from phytosterol, but highest ever reported testosterone yield was achieved using *M. neoaurum* VKM Ac-1815D: 4.59 g/l testosterone was reached from 10 g/l phytosterol thus corresponding to the molar yield of over 66%. The results contribute to the knowledge on phytosterol bioconversion by mycolicibacteria, and are of significance for one-pot testosterone bioproduction from phytosterol bypassing the intermediate isolation of the 17-ketoandrostanes.

**Graphical Abstract:**

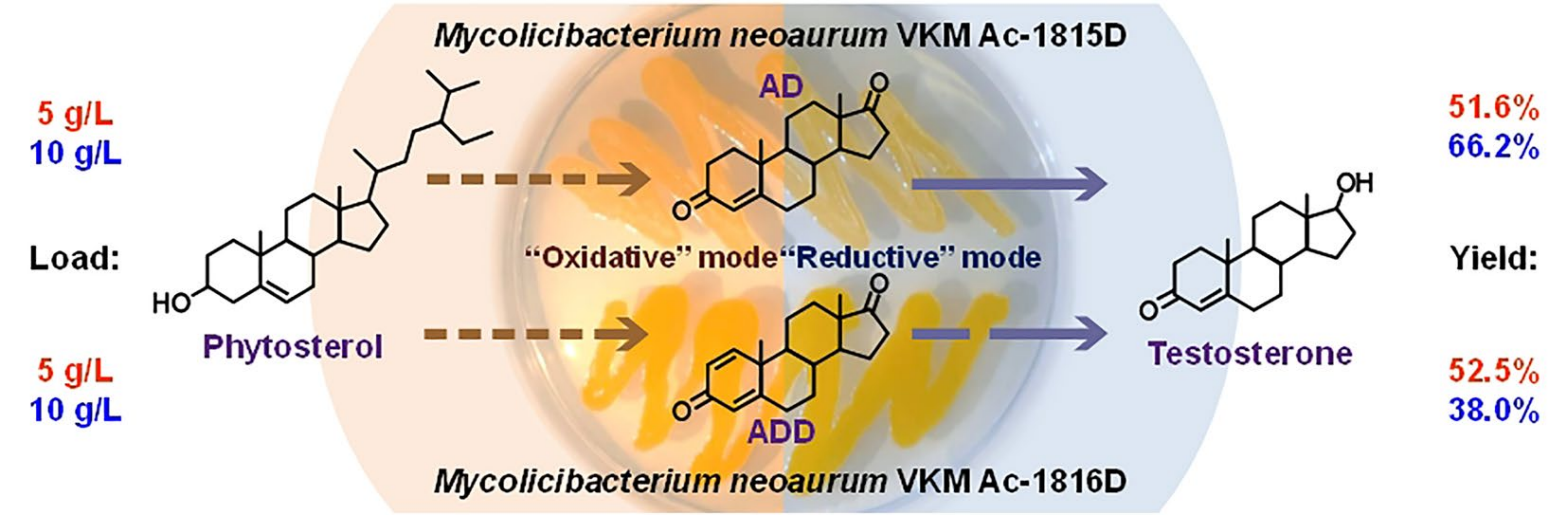

**Supplementary Information:**

The online version contains supplementary material available at 10.1186/s40643-022-00602-7.

## Introduction

Testosterone is the main male sex hormone that plays a role in vital processes in the body. In medicine, the drugs based on testosterone and the derivatives are used to treat endocrinological disorders, metabolic diseases, such as obesity, age-related changes and others (Zhao et al. 2021).

In mammals, testosterone is formed from cholesterol in a cascade of reactions, one of which is catalyzed by 17β-hydroxysteroid dehydrogenases (17β-HSD) and plays an important role in the metabolism of estrogens, androgens, and corticosteroids (Miller 1988). Dysfunction of the 17β-HSD enzyme can lead to cessation of reproduction, the development of cancer, osteoporosis, and Alzheimer disease (Breitling 2001).

The 17β-HSD enzymes were detected in vertebrates, invertebrates, as well as in different bacteria and fungi. It should be noted that most 17β-HSDs reversibly catalyze reduction/oxidation of carbonyl/hydroxyl groups at the C-17 of steroid ring D (Zhao et al. 2021). The direct precursors of testosterone synthesis are androst-4-ene-3,17-dione (AD) and androsta-1,4-diene-3,17-dione (ADD) (Garcia et al. 2012; Al Jasem et al. 2014).

Industrially, testosterone is synthesized chemically (1) from AD using a four-step process (Imada et al. 1981) or (2) from sterols in a multi-step process (Hoberman and Yesalis 1995). However, in addition to other disadvantages, such as environmental risks, chemically synthesized testosterone may cause undesirable side effects (Sood et al. 2017).

Biotechnological production of testosterone is possible from cheap and readily available natural sources—phytosterols, which are a mixture of plant sterols, via the intermediate production of AD, or ADD. Production of AD/ADD from phytosterols is well-reported for actinobacteria, mainly related to *Mycolicibacterium* species, such as *M. neoaurum* (formerly classified as *Mycobacterium neoaurum,* Gupta et al. 2018). These strains are capable of sterol sidechain degradation with simultaneous 3β-ol-5-en- to 3-keto-4-en-moiety modification, and also possess 17β-hydroxysteroid dehydrogenase activity (Liu et al. 1994; Lo et al. 2002; Egorova et al. 2002, 2005). The engineered strains of *M. neoaurum* have been created that effectively produced AD and ADD from phytosterol even at its high concentrations (Shao et al. 2015; Liu et al. 2018; Zhou et al. 2019; Wang et al. 2019).

Production of testosterone from cholesterol and phytosterols by the wild-type and engineered mycobacterial strains has been reported earlier (Lo et al. 2002; Egorova et al. 2009; Karpov et al. 2016; Strizhov et al. 2016; Fernandez-Cabezon et al. 2017).

In the testosterone-producing *Mycobacterium* sp. Et1 strain derived from *Mycobacterium* sp. VKM Ac-1815D (syn. *Mycolicibacterium neoaurum* VKM Ac-1815D) two 17β-HSDs have been identified. One of them was accounted for the bidirectional oxidation–reduction of the oxygen function at C17, while the second one irreversibly oxidized 17β-alcohols to the corresponding 17-ketones (Egorova et al. 2002). Later, irreversible oxidation of testosterone to AD and boldenone (1(2)-dehydro-testosterone, dTs) to ADD has been reported for Hsd4A which was annotated as 17β-HSD in *M. neoaurum* ATCC 25795 (Xu et al. 2016). The orthologous *hsd4A* genes have been identified also in the genomes of *M. neoaurum* VKM Ac-1815D and 1816D (Shtratnikova et al. 2014; Bragin et al. 2013a, 2020). Interestingly, the engineered mycobacteria strains with overexpression of *hsd4A* demonstrated higher AD and ADD production (Wang et al. 2019; Liu et al. 2018, respectively). Thus, production of testosterone by the wild type mycobacterial strains is highly likely due to the activity of the reversible 17β-HSD (Egorova et al. 2002).

Presently, the level of testosterone microbial production from cholesterol is relatively small: its molar yield does not exceed 52% even at low cholesterol loads (no more than 1 g/L) (Liu et al. 1994; Liu and Lo 1997; Borrego et al. 2000; Kumar et al. 2001; Fernandez-Cabezon et al. 2017). The transformation of phytosterols to testosterone has been reported for *Mycobacterium* sp. (Lo et al. 2002) and *Mycobacterium* spp. VKM Ac-1815D and VKM Ac-1816D (Egorova et al. 2009). It should be noted that based on the results of whole genome sequencing and phylogenetic analysis the strains VKM Ac-1815D and 1816D were re-classified as the *M. neoaurum* species (Bragin et al. 2013b; Shtratnikova et al. 2014). In the above studies, the level of testosterone production from phytosterol was reported to be still insufficient for industrial application.

The composition of the nutrient media used significantly affects the efficiency of sterol bioconversion to AD/ADD and their further biotransformation to testosterone (Donova et al. 2007). Due to the poor water solubility of sterols and the toxicity of both the substrate and the product to microorganisms, the biotransformation of sterols is somewhat limited (Fernandez et al. 2003). Cyclodextrins (CDs) can improve productivity of steroids biotransformation by increasing conversion rate, conversion ratio, or substrate availability to the microbial enzymes involved (Fernandez et al. 2003; Donova et al. 2007; Shen et al. 2012; Zhou et al. 2019; Su et al. 2020). This enhancement occurs due to increased solubility of sterols through the formation of the complexes with CDs (Khomutov et al. 2002; Ma et al. 2009), or, on the other hand, could be associated with the reduction of inhibitory effect of the steroid metabolites (Perez et al. 2006; Su et al. 2020) and even up-regulation of most proteins involved in sterol metabolism (Su et al. 2020).

Ensuring the necessary pool of electron donor cofactors such as NADH and NADPH has been reported to be a key limitation in the use of 17β-HSD (Peltoketo et al. 1999; Fogal et al. 2010; Ding et al. 2021). This bottleneck can be overcome through the optimal addition of exogenous glucose, which is an additional substrate for NADH regeneration. Indeed, the molar yield of testosterone or dTs produced by the strains of *Mycobacterium* (syn. *Mycolicibacterium*) was increased when feeding by glucose alone or in combination with lactose (Llanes et al. 1995; Liu and Lo 1997; Lo et al. 2002; Tang et al. 2021). In addition, as shown earlier, the accumulation of testosterone from AD depends on the concentration of dissolved oxygen (Lo et al. 2002).

Herein, we studied phytosterol conversion to testosterone at high substrate loads (up to 10 g/l) by the AD-producing strain of *M. neoaurum* VKM Ac-1815D and ADD-producing strain of *M. neoaurum* VKM Ac-1816D. As shown, the genomes of the two strains are almost identical, but among 13 SNPs revealed, one is in the *kstD* gene (corresponds to the replacement of Leu135 to Ser135) that results in preferable accumulation of ADD instead of AD in *M. neoaurum* VKM Ac-1816D (Bragin et al. 2013a, 2020). The strategy for optimizing “one-pot” production of testosterone from phytosterol (without intermediate AD/ADD isolation) is based on the idea that oxidative cleavage of the phytosterol side chain and reduction of the 17-carbonyl group require different Red/Ox conditions. The effect of medium composition, regimen of glucose supplement and aeration were studied to ensure high level of testosterone bioproduction bypassing intermediate isolation of 3,17-diketosteroids, such as AD and ADD.

## Materials and methods

### Reagents

Soybean phytosterol (total sterols—95.47%: including β-sitosterol—42.39%, campesterol—23.48%, stigmasterol—26.08%, brassicasterol—3.52%) was purchased from Jiang Su Spring Fruit Biological Products Co, LTD (China). AD, ADD, testosterone, dTs were obtained from Steraloids (USA). 20-hydroxymethylpregn-4-en-3-one (HMP) (89% purity) and 20-hydroxymethylpregna-1,4-dien-3-one (HMPD) (95% purity) were obtained from the MTOC Laboratory of Skryabin Institute of Biochemistry and Physiology of Microorganisms, RAS (IBPM RAS).

Randomly methylated β-cyclodextrin (mCD) CAWASOL W7 M1.8 was purchased from Wacker Chemie (Germany), corn extract—from Sigma (USA), sucrose palmitate stearate 15 (SPS)—from Serva (Germany), NADH and NADPH—from Panreac AppliChem (Spain). Full fat soy flour was obtained from Soja PAN (Austria). Other materials were of reagent grade and purchased from domestic companies.

### Microorganisms and cultivation

The strains of *M. neoaurum* VKM Ac-1815D and *M. neoaurum* VKM Ac-1816D were obtained from the All-Russian Collection of Microorganisms (VKM IBPM RAS). The strains were cultured at 30 °C aerobically (220 rpm) as described earlier (Donova et al. 2007).

### Assay of 17β-HSD and 1-ene-reductase activity in vitro

The cells were harvested by centrifugation at 8000×*g*, washed twice with cold 0.02M potassium phosphate buffer and frozen at – 70 °C. The washed frozen cells (7 g) were disrupted by a single passage through French press, and then suspended in 10 ml of 15 mM Tris-HCl buffer with 150 mM NaCl, pH 8.0. Cell debris was removed by centrifugation at 30,000×*g* for 2 h at 4 °C. Reaction mixture contained TRIS-HCl buffer (pH 8.0), 20 mM NaCl, 600 μM NADH (or NADPH), 118 μM AD or ADD, and 500 μl of supernatant or 350 mg of cells debris (as mentioned below). The total volume of the reaction mixture was 3 ml. AD and ADD transformation was carried out on a rotary shaker at 180 rpm (agitation conditions) or without mixing (steady conditions) for 20 h at 37 °C. The transformation products were twice extracted with excessed ethyl acetate, evaporated, re-solved in 200 µl of ethyl alcohol and analyzed using TLC (thin layer chromatography) or HPLC (high pressure liquid chromatography) as described below.

### Phytosterol transformation by growing cells

The nutrient medium M1 for phytosterol bioconversion was consisted of (g/l): glucose (10–20); urea (0.25), corn extract (10); CaCO_3_ (3); SPS 0.25), MgSO_4_·7H_2_O (0.2); FeSO_4_·7H_2_O (0.005); ZnSO_4_·7H_2_O (0.002), 0.02 M Na-phosphate buffer (pH 7.0). The medium M2 additionally contained 10 g/l soy flour. In some experiments, glucose was replaced with glycerol (10 g/l), and mCD was added at a molar ratio to phytosterol ranging from 0.3 to 1.4. If not otherwise mentioned, glucose (5 g/l) was added daily. Phytosterol (5–20 g/l) was added to the media before sterilization (0.5 atm, 30 min), as a powder, or as a suspension with SPS and CaCO_3_. For inoculation, 20% (v/v) of the seed culture grown as described in 2.2 was used.

Biotransformation was conducted in 750 ml Erlenmeyer flasks containing 100 ml of culture broth at 30 °C under “oxidative” mode (aerobically on a rotary shaker at 220 rpm) or successively under “oxidative” and then “reductive” modes. For the microaerophilic conditions (or “reductive” mode) the stirring was slowed down twice (to 100 rpm) when residual molar phytosterol content decreased to 10% of the initial concentration.

### Steroids analyses

The samples (1 ml) were taken at least once a day. The steroids were extracted and assayed by TLC and HPLC as described earlier (Lobastova et al. 2021).

### Cell growth assay

For CFU counting, the samples were serially diluted with saline under vigorous agitation and plated on the solid medium for inoculum growing supplied with agar (20 g/l).

### Reduced sugars concentration

Estimated in accordance with (Gusakov et al. 2011). Optical density was measured using a 2-beam spectrophotometer (Shimadzu, Japan) at 600 nm. Glucose concentration was calculated using the calibration curve.

### Statistical analysis and calculations

The efficiency of phytosterol transformation (%) was calculated as the total molar concentration of all steroidal products divided by initial molar phytosterol concentration multiplied by 100%.

Molar product yield (%) of the transformation metabolites was estimated as: molar concentration of the evaluated product divided by molar concentration of substrate (phytosterol) multiplied by 100%.

All the experiments were performed 3–5 times and the data were statistically analyzed by one-way ANOVA. Standard deviation is shown as error bars at figures and as the variation of mentioned values after “±” sign. At Figs. 4B, 6A, B (ADD, HMP+HMPD, dTs values), and Table 1 errors were not more than 3–7%, and not shown for better data presentation.

**Table 1:** Products of AD and ADD transformation by cellular fractions of *Mycolicibacterium neoaurum* strains

Cellular fraction	*M. neoaurum* VKM Ac-1815D				*M. neoaurum* VKM Ac-1816D			
	Substrate							
	AD	AD + NAD(H)	ADD	ADD + NAD(H)	AD	AD + NAD(H)	ADD	ADD + NAD(H)
a. Steady incubation conditions								
Supernatant	Ts^1^ (8.6%^2^)	Ts (19.4%)	dTs (6.9%) AD (13.8%)	dTs (21.2%) AD (6.3%)	Ts (3.1%)	Ts (22.7%)	dTs (4.0%)	dTs (20.1%)
Cellular debris	Ts (7.0%)	Ts (12.7%)	dTs (7.4%) AD (13.4%)	dTs, tq^3^ AD (12.4%)	Ts, tq ADD (12%)	Ts (9.7%) ADD (4.2%)	AD (78.5%)	AD (22.9%)
b. Agitation incubation conditions								
Supernatant	Ts, tq	Ts (25.2%)	AD, tq	dTs (37.3%) AD, tq	Ts, tq ADD, tq	Ts (17.5%) ADD (10.0%)	dTs, tq AD, tq	dTs (21.5%) AD (10.8%)
Cellular debris	Ts, tq	Ts (21.6%) ADD (12.7%)	AD, tq	AD (96.0%)	Ts, tq ADD, tq	Ts (12.9%) ADD (8.8%)	nd^4^	AD (97.0%)

^1^Ts - testosterone, androst-4-en-3-on-17-ol, ^2^molar yields of transformation (%) are indicated; ^3 ^tq - trace quantity; ^4^nd - not detected

## Results and discussion

### 17β-HSD and 1-ene-reductase activities and localization

In our previous works the presence of 17β-HSD activity has been demonstrated for *M. neoaurum* Ac-1815D and its related strains, the mutant Et1 and *M. neoaurum* VKM Ac-1816D (Egorova et al. 2002, 2005, 2009). As shown for *M. neoaurum* Et1, the 17β-HSD is located mainly in the peripheral cytoplasmic zone of the cells adjoining the cytoplasmic membrane (Egorova et al. 2005).

**Fig. 1:**
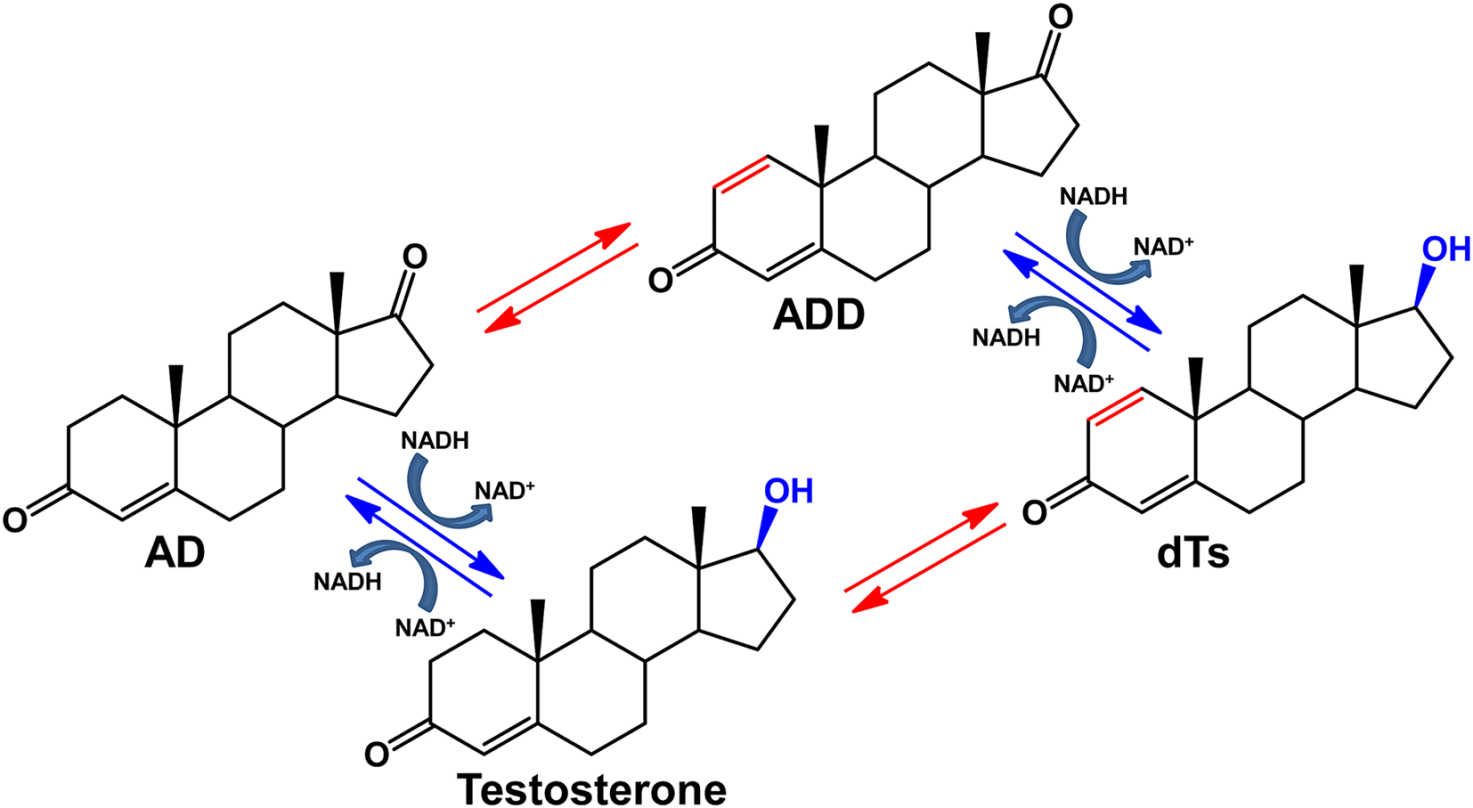
Scheme of C_19_ steroid compounds transformation: red arrows—hydrogenation/dehydrogenation at C1(2). Blue arrows—reduction/oxidation at C17

In this study, we estimated AD and ADD transformation *in vitro* by the cellular fractions of the strains 1815D and 1816D under different aeration conditions and NAD(H) supplement. The observed reduction and oxidation reactions of the steroid core at C17 and C1(2) is summarized in Fig. 1.

As shown in Table 1, testosterone was formed as the only, or major product from AD by both cytosol and membrane fractions of *M. neoaurum* VKM Ac-1815D. The testosterone amounts increased in the presence of NAD(H) being higher under the aerobic conditions (21.6–25.2% (mol.)). ADD was observed among the products in the debris fraction, thus indicating the presence of 3-ketosteroid-1-dehydrogenase (KstD) activity toward AD (Table 1). In fact, the presence of this activity is not only demonstrated *in vitro*, but also its role has been demonstrated *in vivo* in the *M. neoaurum* VKM Ac-1816D strain ("Production of testosterone by* Mycolicibacterium neoaurum* VKM Ac‑1816D” and “Transformation of phytosterol by* Mycolicibacterium neoaurum* VKM Ac‑1816D under “oxidative” and “reductive” modes”). The existence of 1(2)-en reductase can be fundamental to explain the metabolism of sterols in *Mycolicibacteria*, but requires further confirmation

When using ADD as a substrate, AD (mainly) and dTs were formed under microaerophilic conditions. The amounts of dTs formed in the soluble fraction significantly increased both under microaerophilic and aerobic conditions in the presence of NAD(H), while the major product from ADD in the cellular debris was AD. Notably, the conversion of ADD to AD in the cell debris fraction reached 96% (mol.) when adding NAD(H) (Table 1).

Unlike *M. neoaurum* Ac-1815D, the strain of *M. neoaurum* Ac-1816D converts phytosterol mainly to ADD under aerobic conditions (Bragin et al. 2013b). As follows from Table 1, both cellular fractions of the strain 1816D also possessed 17β-HSD activity that was evidenced by AD→testosterone, and ADD→dTs conversions. Higher amounts of 17β-alcohols were expectedly accumulated with the addition of NAD(H).

The results confirmed the presence of 17β-HSD (AD→testosterone; ADD→dTs), 1-ene reductase (ADD→AD) and 3-KstD (AD→ADD) activities in both strains. Expectedly, 3-KstD activity was higher in the case of 1816D cell fractions, and ADD was formed from AD along with testosterone both under microaerophillic and aerobic conditions, while the fractions of 1815D strain provided more selective testosterone production from AD (Table 1). The presence of both 3-kstD and 1(2)-en reductase has been reported earlier for Mycobacterium globiforme 193 (later re-classified as *Arthrobacter globiformis* 193 and further—as *Nocardioides simplex* VKM Ac-2033D) (Shtratnikova et al. 2021). Unlike 3-KstD, purified 1-en-reductase was active in the presence of NADP(H) (Lestrovaya and Bukhar 1970).

In both cases, transformation level of ADD was not significantly different from that of AD, but taking into account lower AD solubility and differences in the toxicity of AD and ADD, the latter could be preferred substrate. Selectivity of the 17β-alcohols production increased under microaerophilic conditions being higher for the cytosol fraction of 1816D strain. The most active hydrogenation of C_1_–C_2_-double bond (ADD→AD conversion of 96% and 97%, mol.) was observed for the membrane fractions in the presence of NAD(H) at high aeration. Noteworthy, NAD(H) negatively affected 1-ene-reductase activity associated mainly with the membrane fraction (Table 1). NADP(H) did not have a positive effect on the 17β-HSD activity for both supernatant and debris fractions of both studied strains (data not shown).

Thus, the obtained data confirmed intracellular localization of 17β-HSDs in both *M. neoaurum* VKM Ac-1815D and Ac-1816D; the 17β-HSD of *M. neoaurum* VKM Ac-1816D and VKM Ac-1815D is represented by a soluble and membrane-bound forms. The presence of NAD(H) is a condition for the high 17β-HSD activity, as also evidenced by literature data (Liu and Lo 1997; Fogal et al. 2010; Benach et al. 2002). On the contrary, NADP(H) has been shown to be the main source of reducing equivalents for *Mycobacterium* sp. NRRL 3805, while only 10–15% of activity was observed in the presence of NAD(H) (Goren et al. 1983).

Soluble and membrane-bound 17β-HSDs have been also reported for other microorganisms. The 17β-HSD activity of the mutant strain *Mycolicibacterium* sp. Et1 was found both in the cytosol-soluble and membrane-bound form. By the method of cytochemical reaction it has been shown that 17β-HSD is located in the periphery zone of cytoplasm and poorly associated with the cell membrane (Egorova et al. 2002). 17β-HSDs of *Pseudomonas testosteroni* are located on external side of the cytoplasmic membrane (Lefebre et al. 1979). The main part of 17β-HSDs of *Mycolicibacterium* sp. NRRL 3805 was detected predominantly in the membrane-mesosomal fraction (Goren et al. 1983). A comparative study of the AD and ADD transformation by washed *Mycobacterium* sp. NRRL B-3683 cells showed that testosterone is formed only from ADD (Hung et al. 1994). In this study, the effect of NAD(H) and aeration conditions on the 17β-HSD activity of mycolicibacteria cell fractions was shown for the first time.

### Phytosterol bioconversion by *M. neoaurum* VKM Ac-1815D: the “oxidative” mode

A series of preliminary experiments were conducted that included the evaluation of various carbon and nitrogen sources, surfactants and solubilizing agents in the bioconversion of phytosterol by *M. neoaurum* VKM Ac-1815D (data not shown). As a result, a complex nutrient medium M1 was chosen that provided active culture growth and a time-coordinated conversion of phytosterol to AD.

**Fig. 2:**
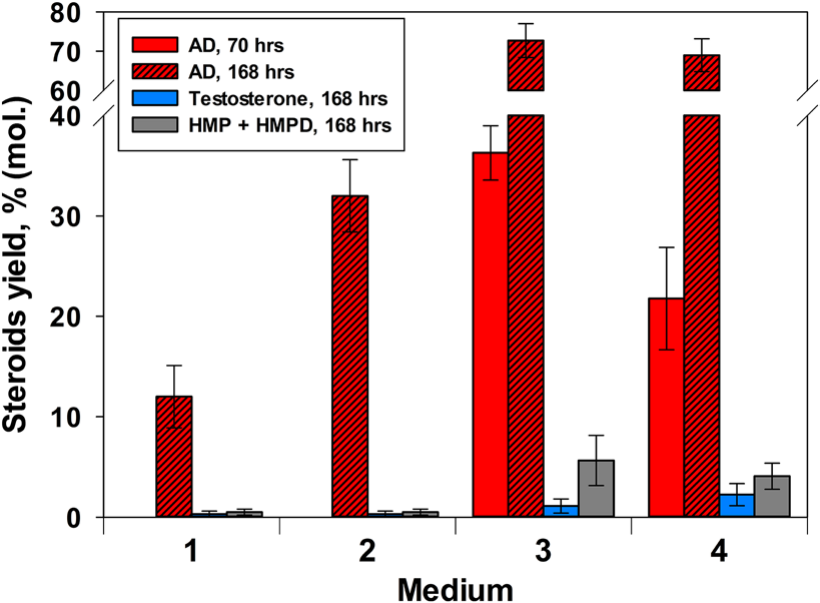
Influence of media composition on phytosterol^1^ biotransformation by *Mycolicibacterium neoaurum* VKM Ac-1815D under “oxidative” mode. 1—Medium M1 + 10 g/l glucose; 2—medium M2 + 10 g/l glucose; 3—medium M2 + mCD + 10 g/l glucose; 4—medium M2 + mCD + 20 g/l glucose. ^1^Initial phytosterol load was 10 g/l. The molar ratio of phytosterol to mCD was 1:0.3. Medium M2 is the medium M1 supplemented with the soy bean flour

As follows from Fig. 2, the supplement of the medium M1 with fat soy flour (medium M2) provided at least 2.7-fold enhancement of phytosterol to AD bioconversion. Increasing of the soy flour concentration up to 20 g/l, replacement or combining the soy flour with lupine or cotton flours (20 g/l or 10 g/l of each) had little effect on the yield of AD (data not shown).

The bioconversion of phytosterol to AD was significantly enhanced in the presence of mCD: an almost sevenfold increase in the output of AD—up to about 73% (mol.) (over 5 g/l) was observed when mCD was added to the M2 containing soy flour (Fig. 2: 1 vs. 3).

The data are in accordance with a well-known CD-mediated enhancement of phytosterol conversion by mycobacteria (Donova et al. 2007; Shen et al. 2012; Zhou et al. 2019). The multiple mechanism of the CD action includes steroid solubilization due to formation of inclusion [steroid–CD] complexes (Khomutov et al. 2002; Ma et al. 2009), alterations in the cell wall permeability for steroids and nutrients (Donova et al. 2007), but also CDs may affect steroid-transforming enzymes and expression of the genes involved in the steroid catabolic pathway (Shtratnikova et al. 2017; Su et al. 2020), and reduce the inhibitory effect of the products (Perez et al. 2006).

When glycerol was applied instead of glucose in the medium M1, phytosterol to AD bioconversion decreased slightly (data not shown). Doubling the concentration of glucose in the medium M1 had a negative effect on the initial rate of bioconversion and the final output of AD: after 70 h of bioconversion, the content of AD was 1.7 times lower when using 20 g/L compared to 10 g/l as the initial concentration of glucose (Fig. 2: 3, 4—red columns). Noteworthy, low amounts of testosterone were detected when medium M1 was used under the conditions of the intensive stirring (“oxidative” mode). Higher initial content of glucose in the medium M2 resulted in the elevated production of testosterone (Fig. 2: 3, 4—blue columns). Similar effects were observed at the increased glycerol concentration (20 g/l), or glycerol plus glucose usage (10 g/l of each) (data not shown). Therefore, in the poor of carbon media (M1 without soy flour) and in the beginning step of the oxidative transformation (phytosterol → AD) an excess of energy and carbon source could be unfavorable for the degradation of aliphatic side chain of phytosterol that gives ATP and NAD(H). On the contrary, when using rich media (such as M2) additional glucose is favorable for the transformation of AD to testosterone, since it shifts the redox balance toward reduction.

The yield of AD (68.97–72.71% (mol.)) from 10 g/l phytosterol achieved in this study using medium M2 and low mCD content is generally comparable with that reported earlier for the same strain when much higher mCD concentration was used (Donova et al. 1997, 2007). Moreover, the total content of the undesirable C22-steroids—HMP and HMPD (Figs. 2: 3, 4—grey columns) was 6% (mol.), i.e., 2–2.5 times lower as compared with that reported earlier for the same strain when using higher mCD concentrations (Donova et al. 1997, 2007). Possibly this fact could be explained by a decrease of mCD concentration and corresponding [C22-steroids–mCD] complexes, respectively.

So, the addition of the full-fat soy flour to the medium allows achieving about the same AD yield at the lower mCD content, but during extended incubation period. The results are of importance taking into consideration relatively high cost of the mCD.

Thus, the use of soy flour as a component of the media for phytosterol bioconversion provided effective accumulation of AD and a decrease in the level of by-products. Perhaps the described effect of fully fat soy flour is related to its properties, such as the presence of insoluble small particles, organic nitrogen and fat/oily components. It is favorable not only for the growth of culture, but also for phytosterol bioconversion. Small particles in a flour may serve as sorbents for hydrophobic mycolicibacteria cells and steroids. High fat and oily components possibly enhance dissolution and distribution of hydrophobic steroids; organic nitrogen compounds are additional sources of nitrogen for cell growth. The two last proposals correlate with the fact that lupine and cotton flours additions, that are not rich in amino acids and are not the full-fat, had insignificant influence on phytosterol transformation.

### “One-pot” phytosterol transformation to testosterone by *M. neoaurum* VKM Ac-1815D under different aeration modes

As follows from the abovementioned results (“17β‑HSD and 1‑ene‑reductase activities and localization”), effective regeneration of NAD(H) and decreased aeration are the two major factors influencing reduction of 17-carbonyl group of AD and ADD. These results and literature data (Llanes 1995; Liu and Lo 1997; Peltoketo et al. 1999; Lo et al. 2002; Tekucheva et al. 2022) indicate that effective phytosterol bioconversion to AD/ADD and accumulation of testosterone/dTs require different intracellular Red/Ox status, i.e., cannot be combined in time.

Therefore, the following approaches were suggested to improve 17β-alcohols production from phytosterol: (i) switching the process from oxidizing (intensive aeration conditions) to reducing (microaerophilic conditions) after phytosterol side chain oxidation completion; ii) fractional addition of glucose into the complex medium to provide the effective regeneration of NAD(H) and high 17β-HSD activity in whole cells.

On the contrary, to improve the efficiency of phytosterol to AD transformation it is necessary to increase NAD^+^ to NADH ratio, and some successful approaches have been developed. For example, heterologous expression of NADH oxidase from *Lactobacillus brevis* and the overexpression of C27-monooxygenase (involved in the phytosterol side chain degradation and also in the NAD^+^ production) allows to increase AD(D) yield up to 94% (mol.) from 5 g/l phytosterol (Su et al. 2017) and 96% (mol.) (1.98 g/l from 3 g/l phytosterol) (Su et al. 2018), respectively. The addition of 9 g/l nicotinic acid (precursor of NAD) also enhanced NAD+/NADH ratio and led to a 37.5% increase in AD yield up to 0.88 g/l from 5 g/l phytosterol (Su et al. 2017).

The elevated concentration of dissolved oxygen in the medium negatively affected the process of AD to testosterone transformation by AD-producing ST2 mutant of *Mycolicibacterium* sp. B-3805S strain, but at the same time, provided an effective conversion of phytosterol to AD (Lo et al. 2002).

The results of phytosterol transformation by *M. neoaurum* VKM Ac-1815D and 1816D exploiting two different aeration and glucose supplement regimens are shown in “One‑pot” phytosterol transformation to testosterone by *M. neoaurum* VKM Ac‑1815D under different aeration modes” and “Production of testosterone by* Mycolicibacterium neoaurum* VKM Ac‑1816D” secctions, respectively. As a first step, the influence of the fractional glucose additions to the medium M2 on phytosterol bioconversion by *M. neoaurum* VKM Ac-1815D was estimated.

**Fig. 3:**
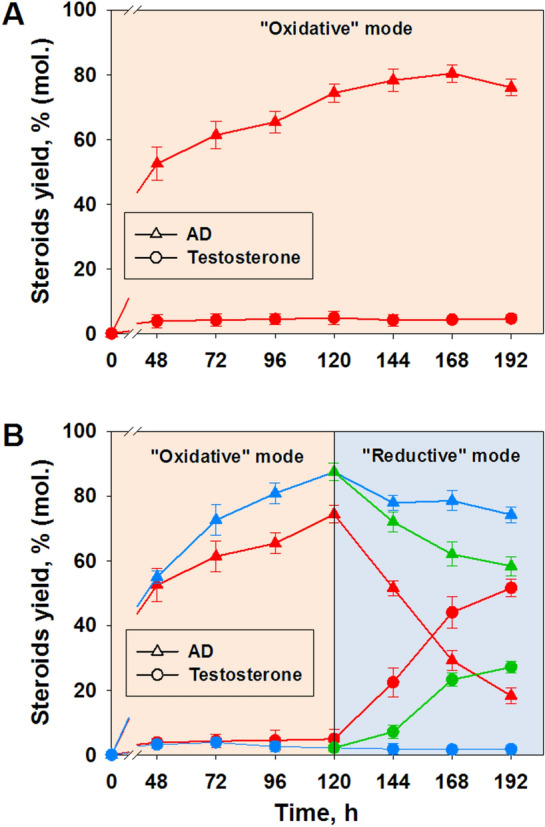
Effect of aeration modes and their combination on phytosterol transformation by *Mycolicibacterium neoaurum* VKM Ac-1815D^1^. **A** Transformation was carried out only under the “oxidative” mode; **B** change of the transformation mode from “oxidative” to “reductive” was conducted at 120 h (vertical line). Red graphs—glucose was added daily throughout transformation period (24–196 h); blue graphs—glucose was not added daily; green graphs—glucose was added throughout “reductive” mode (144–196 h). ^1^The initial phytosterol load was 5 g/l. The molar ratio of phytosterol to mCD was 1:0.3. Glucose (10 g/l) was added to the medium M2 at the inoculation moment (0 h) in all cases and additionally as mentioned in legend

When glucose was added before inoculation and then daily during phytosterol bioconversion under aerobic conditions (“oxidative” mode), the content of AD increased to 76–80% (mol.), while the molar yield of testosterone stabilized at a low level (less than 5%) (Fig. 3A).

When switching from an oxidative to a reductive mode after almost complete phytosterol transformation (~120 h), a significant accumulation of testosterone was observed (Fig. 3B). The dynamics of its accumulation depended on the glucose supplements regimen.

We applied three options: (i) without daily glucose addition (control), (ii) daily glucose (5 g/l) addition during the whole phytosterol bioconversion period (since 24–196 h), and (iii) daily glucose (5 g/l) addition during the “reductive” mode only (since 120–196 h). In all variants the nutrient medium was supplemented with 10 g/l glucose before inoculation.

In the first case, a slight decrease of AD content was observed (from 87.4% to 74.2% (mol.)), and the testosterone yield did not exceed 2% (mol.) (Fig. 3B, blue graphs). Similar results were reported earlier during the transformation of phytosterol by the same strain in the mineral medium with glycerol (Donova et al. 1997).

When using option ii), a rapid decrease in AD level and the corresponding elevation of testosterone concentration was observed. The testosterone yield increased tenfold to reach 1.79±0.08 g/l (corresponds to the molar yield of 51.60±2.72%) for 192 h. However, more than 18% of AD remained unconverted (Fig. 3B, red graphs). Much lower rate of testosterone accumulation and higher AD content (Fig. 3B, green graphs) were observed under the “reductive” mode when the medium M2 was supplemented with glucose only during “reductive” mode (after 120 h).

Noteworthy, adding glucose throughout the entire transformation process (ii) resulted in higher testosterone production compared to the variant when glucose was added after the completion of the “oxidative” stage (iii). Therefore, the addition of glucose only during the "reductive" mode is not enough to achieve high efficiency of testosterone production.

Glucose supplementation mode played insignificant role. Thus, under the "oxidative" mode without daily addition of glucose, its residual concentration dropped rapidly (Additional file 1: Fig. S1 A: green graph), while daily addition provided a smoother decrease (Additional file 1: Figure S1 A: red graph). From the start of daily glucose addition and aeration mode change (120 h) the patterns of glucose utilization were almost the same. *M. neoaurum* VKM Ac-1815 culture can grow under transformation conditions. The active growth started without lag-phase and lasted up to 48 h (Additional file 1: Figure S1B).

The obtained results are in agreement with the data described for *Mycolicibacterium* sp. NRRL B-3805 demonstrating glucose effect on cholesterol (1 g/l) transformation (Liu and Lo 1997). Positive effects of glucose on the 17β-HSD activity of *M. neoaurum* VKM Ac-1815D are also consistent with the enzymatic activity of the recombinant strains *M. smegmatis* mc^2^ 155 (pHSDCT) and *M. smegmatis* ms^2^ 155 (pHSDCL). When resting cells were used, AD was transformed into testosterone more effectively in the presence of 1% glucose, but not glycerol (Fernandes-Cabezon et al. 2017).

Comparative data on the use of glucose and fructose by *M. neoaurum* JC-12 during the transformation of phytosterol (20 g/l) into ADD showed that fructose, used as the initial source of carbon, accelerated the accumulation of biomass, and eliminated the lag phase during the growth of the culture, while glucose application allowed shorter the transformation period (from 168 to 120 h) and increased the productivity of the target process (Shao et al. 2015). Moreover, glucose supplement also slowed down the undesirable testosterone oxidation, which contributed to its higher yield (Liu and Lo 1997; Egorova et al. 2009). Supplements of glucose and limited aeration were found to be the key factors that provide a shift of the 17β-HSD activity toward reduction of AD+ADD formed by *M. neoaurum* VKM Ac-1815D to testosterone in whole *Nocardioides simplex* VKM Ac-2033D cells during the cascade bioconversion of phytosterol (Tekucheva et al. 2022).

### Effect of mCD on testosterone production by *Mycolicibacterium neoaurum* VKM Ac-1815D

As mentioned above, CDs have a great influence on steroid bioconversions, and the effect depends on the concentration and the type of CDs used (Donova et al. 2007; Shen et al. 2012). CD-mediated intensifications of testosterone/dTs production from AD(D) by *Saccharomyces cerevisiae* (Singer et al. 1991), *Nocardioides simplex* (Tekucheva et al. 2022), *Arthrobacter simplex* CPCC 140451 and *Pichia pastoris* GS115 (Tang et al. 2019), and *Mycolicibacterium* strains (Egorova et al. 2009) have been reported, as well as the enhancement of cholesterol and phytosterol conversion to testosterone by *Lactobacillus bulgarius* (Kumar et al. 2001), and by different strains of *actinobacteria* (Donova et al. 2007; Shen et al. 2012; Shtratnikova et al. 2017; Tekucheva et al. 2022).

**Fig. 4:**
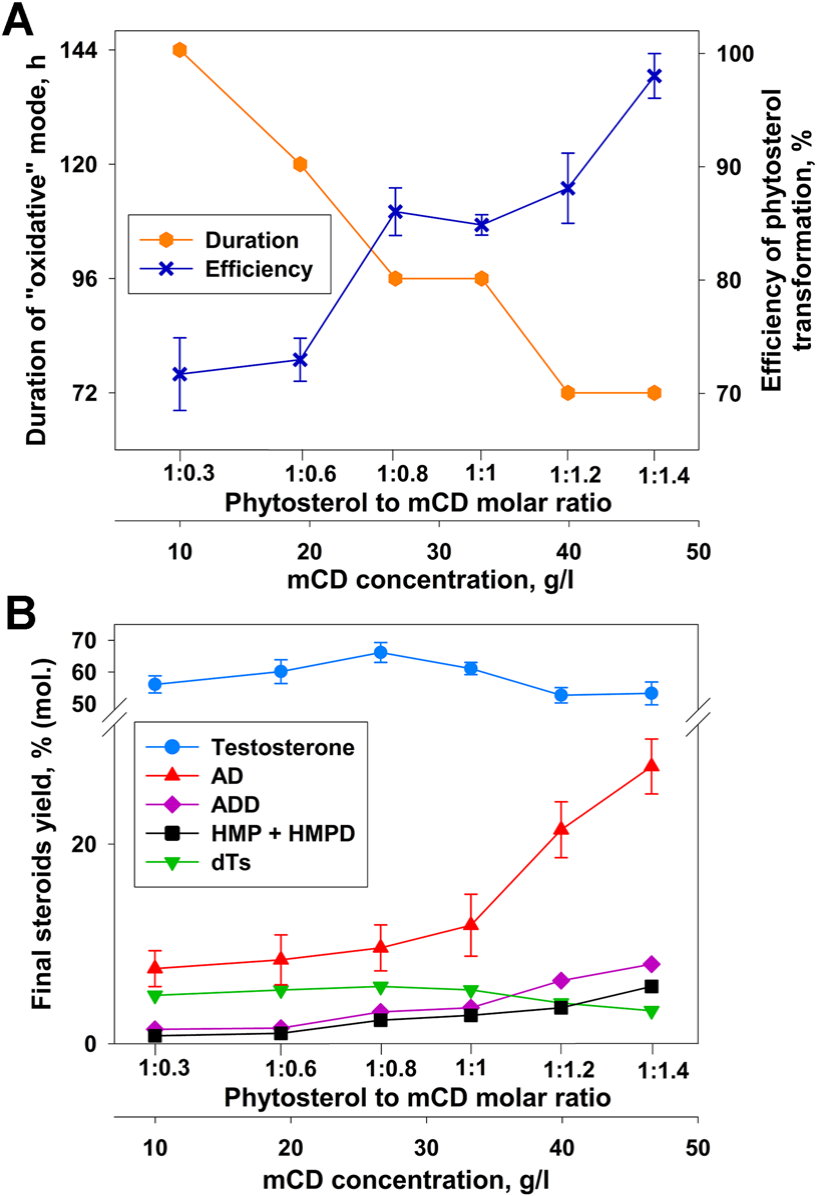
Effect of mCD content on phytosterol transformation by *Mycolicibacterium neoaurum* VKM Ac-1815D^1^. **A** On the process duration and phytosterol transformation efficiency^2^, **B** on the steroids yields. ^1^The transformation was performed in M2 with initial phytosterol load of 10 g/l. At the inoculation moment (0 h) 10 g/l of glucose and further 5 g/l daily was added. The duration of “reductive” mode was 72 h for all phytosterol to mCD molar ratios. The duration of “oxidative” mode was varied. ^2^The efficiency of phytosterol transformation was calculated as total molar concentration of all steroidal products divided by the initial molar phytosterol concentration, %

In this study, an increase in the mCD content from 10 to 47 g/l (corresponds to phytosterol to mCD molar ratio of 1:0.3 and 1:1.4, respectively) favored phytosterol (10 g/l) transformation by *M. neoaurum* VKM Ac-1815D with the contraction of the “oxidative” mode duration from 144 to 72 h (Fig. 4A).

Maximum testosterone level (66.16±3.14% (mol.) or 4.59±0.22 g/l) was reached at the molar phytosterol to mCD ratio of 1:0.8. Decrease in the mCD content resulted in some lower yields of testosterone: 60.14±3.74% and 61.10±1.93% (mol.) were obtained at the molar phytosterol to mCD ratios of 1:0.6 and 1:1, respectively) (Fig. 4B).

As follows from Fig. 4B, the amount of unconverted AD tripled with mCD concentration increase from 10 to 47 g/l, thus indicating that elevated mCD content favors mainly phytosterol conversion, but not AD to testosterone transformation. Noteworthy, the accumulation of other steroid metabolites also increased with elevated mCD concentrations: the content of ADD and C_22_ steroids (HMP + HMPD) increased by 5.5 and 7.2 times, respectively. At the same time, the level of dT decreased with increasing mCD concentration (Fig. 4B). The obtained results made it possible to determine the molar ratio of phytosterol to mCD of 1–0.8 as optimal, since it provided the maximum yield of testosterone with less content of side products and reasonable duration of the “oxidative” mode (Fig. 4).

As reported earlier, no dTs was formed during ADD transformation by *Mycolicibacterium* sp. Et1 in the presence of mCD, while almost full ADD to dTs conversion was observed without mCD (Egorova et al. 2009). It should be noted that AD is less soluble in water as compared with ADD (the solubility of 0.18 mM and 1.63 mM, respectively). AD was shown to form the stronger inclusion complexes with mCD that are characterized lower dissociation constant in comparison with [ADD-mCD] inclusion complex. Low dissociation of the complex may limit the availability of AD to microbial enzymes (Khomutov et al. 2002).

#### Production of testosterone by *Mycolicibacterium neoaurum* VKM Ac-1816D

Conditions that were found to be favorable for phytosterol to testosterone transformation by *M. neoaurum* VKM Ac-1815D (“Phytosterol bioconversion by *M. neoaurum* VKM Ac-1815D: the “oxidative” mode”, ““One‑pot” phytosterol transformation to testosterone by* M. neoaurum* VKM Ac‑1815D under different aeration modes” sections), were applied for phytosterol conversion by *M. neoaurum* VKM Ac-1816D. As mentioned above, unlike AD-producing Ac-1815D, this strain produces ADD as a major metabolite from sterols (Egorova et al. 2002; Donova 2007; Bragin et al. 2013a). *M. neoaurum* VKM Ac-1815D strain does not demonstrate (or demonstrate very weak) KstD activity (“17β‑HSD and 1‑ene‑reductase activities and localization” section) and thus, ADD → AD hydrogenation cannot be explained by a reversible KstD-activity. Apparently, this is due to the fact that the amino acid substitution (Leu135 to Ser135) (Bragin et al. 2013b, 2020) affects the enzyme, which exhibits weak 1(2)-dehydrogenase activity and at the same time the strain may possess sufficiently high 1(2)-reductase activity. The difference in the main steroid metabolites formed from phytosterol was also demonstrated for another strains of *M. neoaurum* (AD for MNR M1 and M3, whereas ADD for MNR) (Shen et al. 2012).

**Fig. 5:**
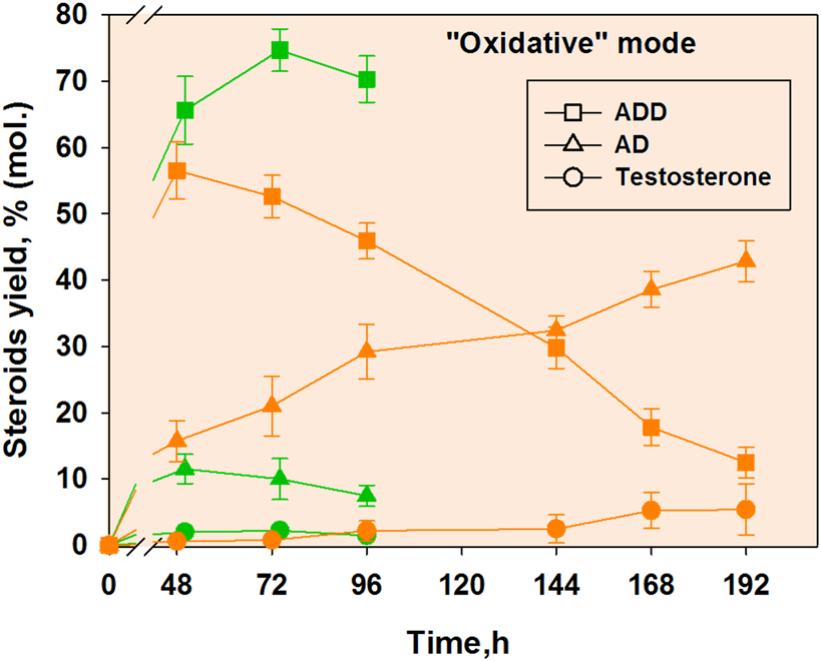
Influence of glucose supplement manner on phytosterol^1^ bioconversion by *Mycolicibacterium neoaurum* VKM Ac-1816D under the “oxidative” mode. Green—single glucose addition (10 g/l), orange—daily glucose feeding. ^1^10 g/l of phytosterol was used; the molar ratio of phytosterol to methylated β-cyclodextrin (mCD) was 1:0.8

In this study, phytosterol (10 g/l) was almost fully converted by the strain 1816D to give 4.95–5.11 g/l ADD (corresponds to molar yield of 72.30–74.67%) under the “oxidative” mode when glucose adding simultaneously with inoculation. Along with ADD, AD was formed from phytosterol and its content slightly decreased after 48 h. The yield of testosterone did not exceed 2.24% (Fig. 5, green graphs).

Different strains of *M. neoaurum* were shown to produce ADD with a high titer ranging from 3.87 to 18.6 g/l from 15 to 40 g/l of phytosterol (Wei et al. 2010; Yao et al. 2013; Shao et al. 2015; Liu et al. 2018).

It should be noted that decrease of the initial pH value from 7.0 to 6.0 did not affected phytosterol bioconversion by *M. neoaurum* VKM Ac-1816D (data not shown). In contrary, as reported for *Mycobacterium* sp. MB-3683 (syn. *Mycolicibacterium neoaurum* MB-3683) the use of pH 6 was more favorable for cholesterol to testosterone bioconversion than pH 7 due to more efficient reduction of AD to testosterone (Borrego et al. 2000).

In our study, the daily glucose feeding resulted in a lower ADD accumulation reached 3.87±0.09 g/l (corresponds to molar yield of 56.53±4.31%) by 96 h. The decrease in the ADD content to 12.49±2.32% (192 h) correlated with more active accumulation of AD (up to 42.87±3.11 %) as well as slower accumulation of testosterone (up to 5.41±3.90 %) (Fig. 5, orange graphs) and dTs (up to 1.41%) (not shown). Total content of HMP and HMPD reached 2.73% for 192 h (not shown). The yield of testosterone and dTs in the case of single glucose addition also was not higher than 5% both for *M. neoaurum* Ac-1815 and 1816D (Figs. 2 and 5). Thus, daily glucose feeding without microaerophilic conditions did not actually contribute to the enhancement of 17β-HSD activity.

A much more active accumulation of AD in comparison with testosterone and dTs conjugated with a decrease of the ADD content under the “oxidative” mode indicates an obvious stimulation of the 1-ene-reductase, rather than 17β-HSD activity by daily glucose feeding to the *M. neoaurum* VKM Ac-1816D culture. Noteworthy, accumulation of testosterone by *M. neoaurum* VKM Ac-1815D under the “oxidative” mode also was not stimulated by daily glucose addition (““One‑pot” phytosterol transformation to testosterone by* M. neoaurum* VKM Ac‑1815D under different aeration modes” section; Fig. 3A), but ADD accumulation was not occurred. All these data correlate with the presence of 1-ene-reductase activity along with 17β-HSD activity as shown in "17β-HSD and 1-ene-reductase activities and localization" section.

As previously reported, glucose feeding stimulated the conversion of ADD to dTs, but not to AD in *Mycolicibacterium* spp. (Llanes et al. 1995), and in washed cells of the mutant *Mycolicibacterium* sp. Et1 (Egorova et al. 2009). A mutant of *Mycobacterium* sp. NRRL B-3805 (syn. *Mycolicibacterium* sp. B-3805) converted cholesterol to testosterone when glucose feeding while cholesterol to AD in the absence of glucose (Liu et al. 1994). Contrariwise, ADD to AD conversion was shown to be stimulated in the presence of mCD, whereas dT was mainly formed in the absence of mCD (Egorova et al. 2009). Hydrogenation of the double C1–C2 bond and simultaneous reduction of the 17-carbonyl group of ADD has been reported also for *Mycobacterium* sp. NRRL B-3683 (syn. *Mycolicibacterium* sp. NRRL B-3683) (Hung et al. 1994). Therefore, the results obtained in this study generally correspond to the literature data and evidence the presence of both 1-ene-hydrogenase and 17β-HSD activities in *M. neoaurum* strains. The prevalence of one or other functionality depends on the Red/Ox conditions and the presence of mCD.

#### Transformation of phytosterol by *Mycolicibacterium neoaurum* VKM Ac-1816D under “oxidative” and “reductive” modes

**Fig. 6:**
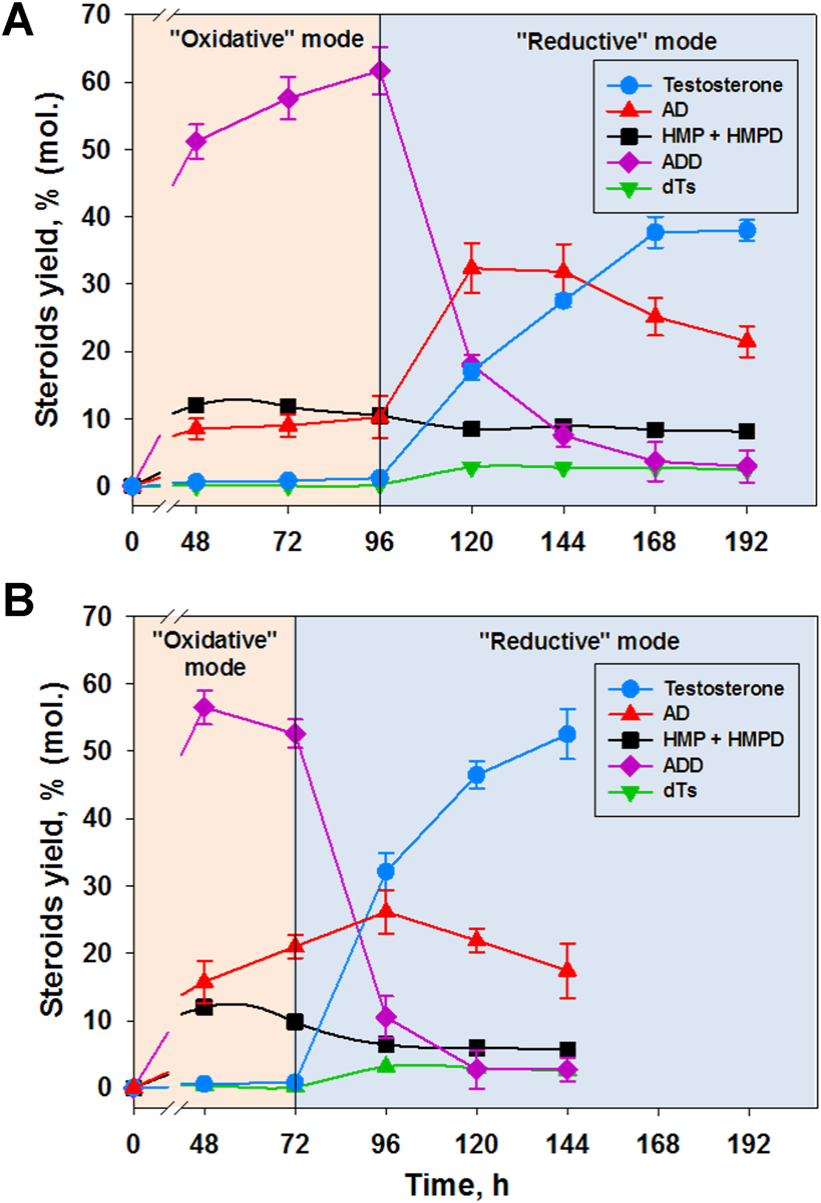
Biotransformation of 10(A) or 5(B) g/l phytosterol into testosterone by *Mycolicibacterium neoaurum* VKM Ac-1816D under two regimens^1^. ^1^the transformation was performed in M2 medium under “oxidative” and “reductive” modes successively with the glucose addition at the inoculation moment (10 g/l) and daily glucose feeding (5 g/l). Molar phytosterol to mCD ratio was 1:0.8

The combination of the “oxidative” (96 h) and “reductive” (96 h) modes and the use of daily glucose feeding ensured an increase in the testosterone yield by 7 times (up to 2.64 ± 0.11 g/l), with an initial phytosterol concentration of 10 g/l (Fig. 6A). Molar yield of testosterone (37.99 ± 1.57%) corresponded to that reported earlier when using 10 times lower substrate concentration (1 g/l) (Liu et al. 1994).

The rate of accumulation of testosterone was approximately the same in the first 3 days of the "reductive" mode (Fig. 6A) and correlated with a decrease in the content of ADD (from around 62% to ~3%), while the rate of ADD consumption during the first 24 h of the "reductive" regimen was clearly higher (Fig. 6A).

So, testosterone accumulates with some delay. AD concentration reached maximum (31.85–32.37%) at the 24–48 h of “reductive” mode (Fig. 6A). This AD dynamic together with the differences in ADD consumption and testosterone accumulation rates suggested that AD was probably the immediate precursor of testosterone in the phytosterol transformation process catalyzed by *M. neoaurum* VKM Ac-1816D cells. Similar dependences have been reported for *Mycolicibacterium* sp. Et1 (Egorova et al. 2002).

At the initial phytosterol concentration of 5 g/l after 72 h under “oxidative” and 72h under “reductive” mode (totally 144 h), the testosterone yield reached 1.83±0.13 g/l (52.52±3.71% (mol.) (Fig. 6B). This molar yield is the highest ever published for *M. neoaurum* VKM Ac-1816D, but less than shown for *M. neoaurum* VKM Ac-1815D–66.16% or 4.59 g/l from 10 g/l phytosterol in this work (“Effect of mCD on testosterone production by* Mycolicibacterium neoaurum* VKM Ac‑1815D” section, Fig. 3).

The total HMP+HMPD molar yield in both variants (phytosterol 5 and 10 g/l) was in the range of 5.64–11.99%, and slightly decreased under “reductive” mode (Fig. 6A, B: 3). The content of dTs in contrast slightly increased after mode changing and varied from 0.3% to 3.2% (Fig. 6A, B: 5).

It should be noted that the mode was changed at the time when the content of residual phytosterol in the medium did not exceed 5–7% (according to TLC data). When 5 g/l of phytosterol was used, the mode was changed after 72 h of cultivation (Fig. 6B, the mode change indicated by a vertical line). If the initial phytosterol concentration was increased up to 10 g/l, the “oxidative” mode was extended by 24 h and lasted 96 h (Fig. 6A). The duration of the “reductive” mode did not depend on the substrate initial concentration.

## Conclusions

“One-pot” biotechnological production of testosterone from available and cheap phytosterols is considered as the most promising approach; however, the corresponding researches are still occasional. In this study, we focused on the search of the conditions favorable for testosterone production by “one-pot” microbial transformation of phytosterol by *Mycolicibacterium* strains.

The curves of major metabolites accumulation during phytosterol transformation indicate that there are two stages characterized by a different dominant enzymatic activity along with the sterol sidechain degrading activity, when the strains of *M. neoaurum* VKM Ac-1815D or 1816D were used (Figs. 2B, 6, respectively):

*First stage. “Oxidative” mode*. Under conditions of intensive aeration, predominant oxidation of the phytosterol side chain and low 17β-HSD activity is observed.

*Second stage. “Reductive” mode*. Under the microaerophilic conditions, active accumulation of testosterone is observed thus evidencing high 17β-HSD activity of the strains. Reduction of 17-carbonyl groups is accompanied with 1-ene hydrogenation (ADD→AD) in the case of *M. neoaurum* 1816D which occurs mainly during the first day after the shift of the “oxidative” to “reductive” mode.

Based on the results obtained, we conclude that (1) the process of the sterols’ side chain degradation and accordingly 17-ketoandrostanes production, and (2) the process of their 17-carbonyl group reduction, proceed under different intracellular Red/Ox status and, therefore, require distinct Red/Ox conditions. Thus, to improve the transformation efficiency of phytosterol to testosterone in the “one-pot” process, it is necessary to create favorable conditions for each “oxidative” and “reductive” modes successively.

An important factor in the industrial biotechnology for the production of steroids is not only the molar yield, i.e., the conversion efficiency of the substrate, but also the final titer, i.e., the amount of product per unit of the medium volume. Increasing the final titer of the product allows the use of a smaller volume of the fermenter and media, reduces the labor and energy costs, and also facilitates and reduces the cost of product isolation and purification. In this work, we increased the substrate load unprecedentedly (4–50 times compared to the published works on phytosterol to testosterone transformation) and at the same time achieved relatively high yields due to optimization of medium composition and physicochemical conditions.

For steroids, it is difficult to overcome the limitations caused by their poor solubility and tendency to form conglomerates at the elevated concentrations that is why it is still impossible to refuse using expensive and hardly recyclable CDs. The combination of the soy flour, mCD and detergent makes it possible not only to reduce the amount of mCD, but also to provide effective phytosterol biotransformation. A more complete conversion of phytosterol into 3,17-diketosteroids was achieved by increasing the active biomass under the "oxidative" mode due to glucose feeding and active aeration. In addition, shift to the “reductive” mode supported the reduction reaction at C17.

The results contribute to the knowledge of physiology of steroid-transforming mycolicibacteria and can be used to create effective biotechnologies for the “one-pot” bioproduction of testosterone from phytosterol bypassing intermediate isolation of 17-ketoandrostanes.

### Supplementary Information

**Additional file 1: Figure S1.** Effect of aeration modes, their combination and glucose supplementation pattern on glucose utilization (**A**) and growth (**B**) of *Mycolicibacterium neoaurum* VKM Ac-1815D during phytosterol transformation^1^. Red—glucose was added daily throughout transformation period (24–192 h), transformation was carried out successively under “oxidative” (24–120 h) and “reductive” (120–192 h) mods^2^; orange—glucose was added daily throughout transformation period (24–196 h), transformation was carried out only under “reductive” mode; green—glucose was added only throughout “reductive” mode (144–196 h), transformation was carried out successively under “oxidative” (24–120 h) and “reductive” (120–192 h) mods^2^. ^1^The initial phytosterol load was 5 g/l. The molar ratio of phytosterol to mCD was 1:0.3. Glucose (10 g/l) was added to the medium M2 at the inoculation moment (0 h) in all cases and additionally (5 g/l) as mentioned in legend. ^2^the change of the transformation mode from “oxidative” to “reductive” was conducted at 120 h (vertical dotted line). ^**3**^ not applicable for orange graph.

## Data Availability

Almost all data analyzed during this study are included in this published article. Another data (mentioned in the article text as “data not shown”) are available from the corresponding author on request.

## Abbreviations

AD: Androst-4-ene-3,17-dione
ADD: Androsta-1,4-diene-3,17-dione
17β-HSDs: 17β-Hydroxysteroid dehydrogenase
CDs: Cyclodextrins
dTs: 1(2)-Dehydro-testosterone
HMP: 20-Hydroxymethylpregn-4-en-3-one
HMPD: 20-Hydroxymethylpregna-1,4-dien-3-one
mCD: Randomly methylated β-cyclodextrin
SPS: Sucrose palmitate stearate
TLC: Thin layer chromatography
HPLC: High pressure liquid chromatography
