# Supplementary figures and images for: Bioproduction of testosterone from phytosterol by Mycolicibacterium neoaurum strains: “one-pot”, two modes
Source: Bioresour Bioprocess. 2022 Nov 4;9(1):116. doi: 10.1186/s40643-022-00602-7 (PMC10992188; doi:10.1186/s40643-022-00602-7)

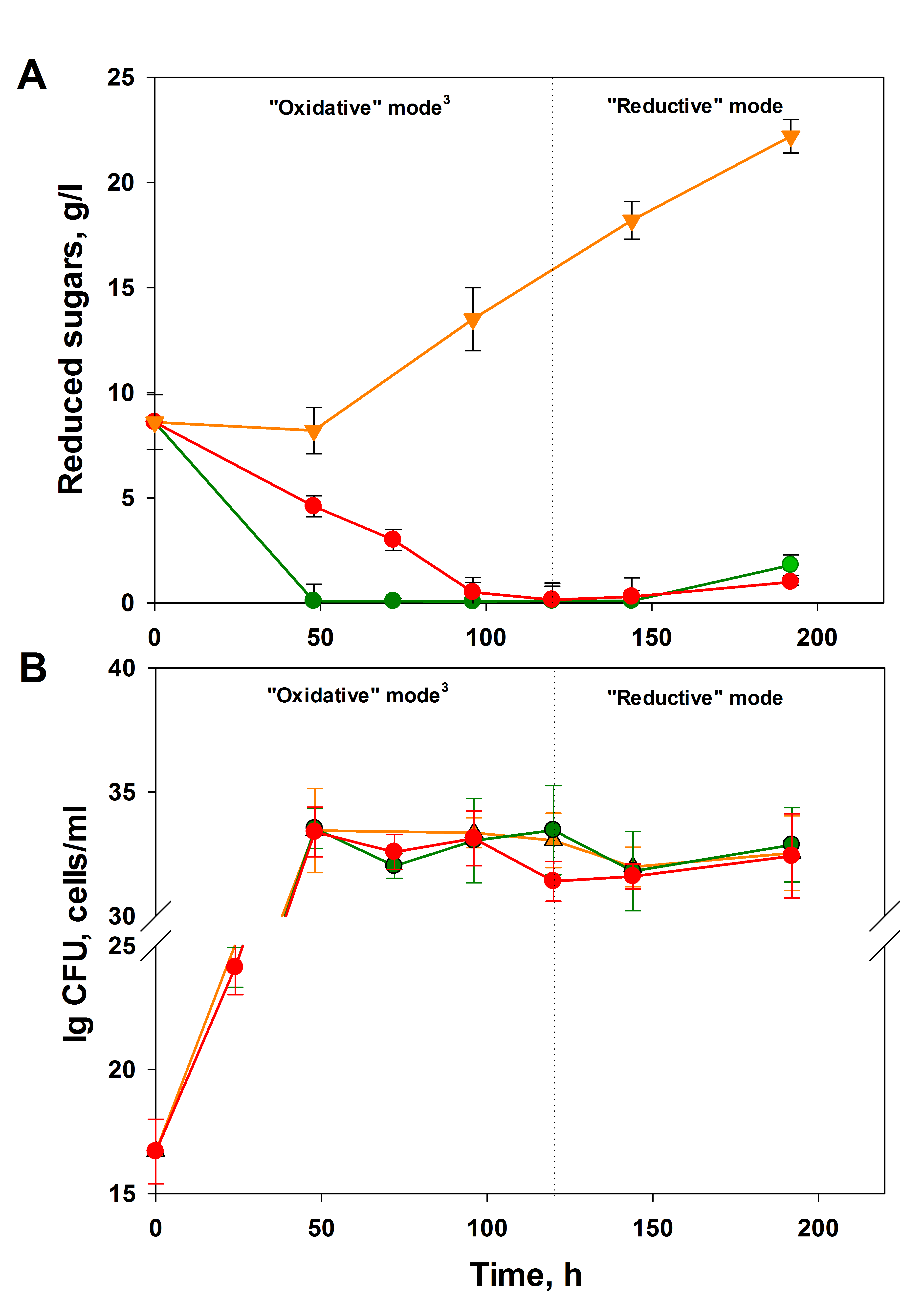

Supplement: Supplementary file 1 — Additional file 1: Figure S1. Effect of aeration modes, their combination and glucose supplementation pattern on glucose utilization (A) and growth (B) of Mycolicibacterium neoaurum VKM Ac-1815D during phytosterol transformation1. Red—glucose was added daily throughout transformation period (24–192 h), transformation was carried out successively under “oxidative” (24–120 h) and “reductive” (120–192 h) mods2; orange—glucose was added daily throughout transformation period (24–196 h), transformation was carried out only under “reductive” mode; green—glucose was added only throughout “reductive” mode (144–196 h), transformation was carried out successively under “oxidative” (24–120 h) and “reductive” (120–192 h) mods2. 1The initial phytosterol load was 5 g/l. The molar ratio of phytosterol to mCD was 1:0.3. Glucose (10 g/l) was added to the medium M2 at the inoculation moment (0 h) in all cases and additionally (5 g/l) as mentioned in legend. 2the change of the transformation mode from “oxidative” to “reductive” was conducted at 120 h (vertical dotted line). 3 not applicable for orange graph. [file 40643_2022_602_MOESM1_ESM.tif]
